# Loss of chromosome 13 material in cellular angiofibromas indicates pathogenetic similarity with spindle cell lipomas

**DOI:** 10.1186/s13000-017-0607-6

**Published:** 2017-02-13

**Authors:** Ioannis Panagopoulos, Ludmila Gorunova, Bodil Bjerkehagen, Kristin Andersen, Marius Lund-Iversen, Sverre Heim

**Affiliations:** 1 Section for Cancer Cytogenetics, Institute for Cancer Genetics and Informatics, The Norwegian Radium Hospital, Oslo University Hospital, P.O.Box 4953, Nydalen, NO-0424 Oslo, Norway; 2 Department of Pathology, The Norwegian Radium Hospital, Oslo University Hospital, Oslo, Norway; 3 Faculty of Medicine, University of Oslo, Oslo, Norway

**Keywords:** Cellular angiofibroma, Cytogenetics, Chromosome 13, Spindle cell lipoma

## Abstract

**Background:**

Cellular angiofibroma is a rare benign mesenchymal neoplasm with morphological and immunohistochemical similarities to spindle cell lipoma. Karyotypic information on cellular angiofibroma is restricted to one case only which showed loss of material from chromosomes 13 and 16. A few other studies using fluorescence in situ hybridization showed deletions of the *RB1* and *FOXO1* loci, both of which are located in chromosome band 13q14. We present here cytogenetic data on two cellular angiofibromas with an abnormal karyotype.

**Methods:**

G-banding and fluorescence in situ hybridization (FISH) analyses were done on two cellular angiofibromas.

**Results:**

In both tumors, a rearrangement leading to loss of chromosome 13 material was seen, together with other structural chromosome abnormalities. FISH analysis showed heterozygous deletion of the *RB1* locus (13q14) in both cases.

**Conclusion:**

Our results demonstrate loss of chromosome 13 material in cellular angiofibroma, though not as the sole cytogenetic change, confirming the (cyto)genetic similarity of these tumors with spindle cell lipomas.

## Background

Cellular angiofibroma is a rare benign mesenchymal neoplasm, first described by Nucci et al. in 1997 in a series of 6 cases that occurred almost exclusively in the vulva of middle-aged women [1]. A few months later, Laskin et al. described 11 histologically similar lesions named “angiomyofibroblastoma-like” tumor, found in the inguinoscrotal area of adult men [2]. Because there were no reproducible morphologic differences between the tumors in females and males, the World Health Organization classification adopted the term “cellular angiofibroma” for this neoplasm in both genders [3]. Although cellular angiofibromas are usually found in the superficial soft tissues of the vulva and inguinoscrotal or paratesticular regions [3], the tumor has also been seen elsewhere such as the retroperitoneum, midtrunk, iliac spine, oral mucosa, knee, upper eyelid, hip, chest wall, axilla, and hypocondrium ([3, 4] and references therein). The etiology is unknown, but expression by tumor cells of estrogen and progesterone receptor suggests that these hormones play a role in tumorigenesis [3]. Cellular angiofibromas display adipose and myofibroblastic differentiation under the influence of hormones, microenvironments, cytokines, and growth factors [1, 2, 5, 6].

The tumors are smaller in women than in men [3], generally well circumscribed, and characterized by two main components: bland spindle cells and small to medium-sized vessels with mural hyalinization [1, 7, 8]. Cytogenetic information about cellular angifibroma is restricted to one case only [9] which by G-banding and fluorescence in situ hybridization (FISH) analyses was found to have the karyotype 45,XY,add(2)(q33),add(12)(p11.2),-13[3]/44,idem,-16[2].ish add(2)(wcp13-),add(12)(wcp13+,LSI 13q14-,LSI 13q34+),der(16)(wcp13+)[3]. Because cellular angiofibroma shares morphological and immunohistochemical similarities with spindle cell lipoma and because monosomy for chromosomes 13 and 16 and unbalanced rearrangements of 13q and 16q are the most frequent aberrations in spindle cell lipomas, the authors suggested that cellular angiofibroma is genetically similar to spindle cell lipoma [9]. In subsequent studies, deletions of the *RB1* and *FOXO1* loci, located in chromosome band 13q14, were found by interphase FISH [7, 10–12].

We present here the cytogenetic data on two cases of cellular angiofibroma. Our results show consistent involvement of chromosome 13 in these tumors with loss of 13q, underscoring the suggested (cyto)genetic similarity between cellular angiofibroma and spindle cell lipomas.

## Methods

### Patients

**Fig. 1:**
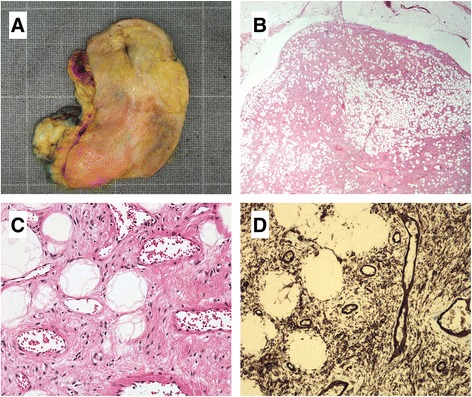
Pathologic examination of the cellular angiofibroma in case 2. **a** Macroscopic picture of the tumor. **b** Low power HE-stained slide showing well circumscribed tumor with adipocytic components. **c** High power HE-stained slide showing spindle cells with admixed adipose tissue and blood vessels. **d** Immunohistochemical analysis demonstrating positivity for CD34

Data concerning patients’ gender and age, tumor location, depth, size, and immunostaining are shown in Table 1. Figure 1 shows the pathologic examination of the cellular angiofibroma of case 2. It includes a macroscopic picture (Fig. 1a), hematoxylin and eosin (HE) staining (Fig. 1b and c), and immunoexpression of CD34 (Fig. 1d). The pathologic findings were similar in case 1. Microscopic examination of the lesions showed well demarcated tumors of spindle cells without atypia with small oval nuclei. In the background, there was a collagenous stroma with many vessels with dilated lumina of different sizes and groups of mature fatty cells. CD34 was positive at immunohistochemical analysis. The morphology was typical for a classical cellular angiofibroma.

**Table 1:** Clinicopathological and cytogenetic data on the cellular angiofibromas

Cases	Sex/Age	Site	Depth	Largest diameter (cm)	Immunohistochemistry	Karyotype
1	M/59	Paratesticular	Subcutaneous	5.5	CD34 Positive	45,XY,add(5)(p13~15),-7,der(13)t(1;13)(q12~21;q14~21),-14,der(16)t(7;16)(q11;q22),del(18)(q21),+mar[9]/46,XY[2]
					S100 Negative	
					ER Negative	
					PGR Negative	
2	M/79	Scrotum	Subcutaneous	7	CD34 Positive	46,XY,t(10;15)(p13;q22),del(13)(q12q22)[15]
					S100 Negative	
					ER Focal positive (20%)	
					PGR Focal positive (10%)

### G-banding, Karyotyping, and FISH

Fresh tissue from a representative area of the tumors was received and analyzed cytogenetically as part of our diagnostic service. The samples were disaggregated mechanically and enzymatically with collagenase II (Worthington, Freehold, NJ, USA). The resulting cells were cultured and harvested using standard techniques. Chromosome preparations were G-banded with Wright's stain (Sigma-Aldrich; St Louis, MO, USA) and examined. Metaphases were analyzed and karyograms prepared using the CytoVision computer assisted karyotyping system (Leica Biosystems, Newcastle, UK). The karyotypes were described according to the International System for Human Cytogenetics Nomenclature [13].

Interphase and metaphase FISH analyses were performed for both cases. The *RB1* deletion probe, purchased from Cytocell (http://www.cytocell.co.uk), was used in order to detect deletion of the *RB1* locus in 13q14.2. It consists of a 318 kb red probe spanning the *RB1* gene and a 13qter green probe acting as a control for chromosome 13. Fluorescent signals were captured and analyzed using the CytoVision system from Leica Biosystems (http://www.leicabiosystems.com/pathology-imaging/cytogenetics/).

## Results

**Fig. 2:**
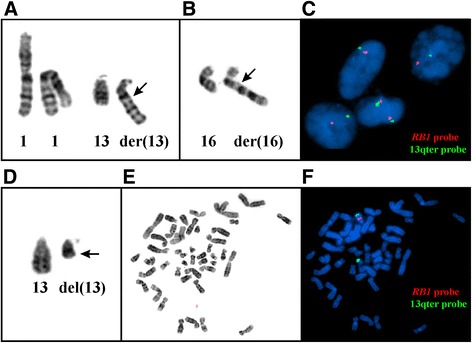
G-banding and FISH information on two cellular angiofibromas. **a**-**c**: Case 1, **d**-**f**: Case 2. **a** Partial karyotype of case 1 showing the two normal copies of chromosome 1, a normal chromosome 13, and the der(13)t(1;13)(q12 ~ 21;q14 ~ 21). **b** Partial karyotype showing the normal chromosome 16 and the der(16)t(7;16)(q11;q22). **c** Interphase FISH showing one red signal (probe for the *RB1* gene) and one green signal (probe for 13qter) in three interphase nuclei and two red and two green signals in one nucleus. **d** Partial karyotype of case 2 showing the normal chromosome 13 and the del(13)(q12q22). **e** G-banding of a metaphase spread used for FISH analysis. **f** FISH results with the *RB1* deletion probe consisting of a 318 kb red probe spanning the *RB1* gene locus and a 13qter green probe acting as a control for chromosome 13. The *RB1* locus was deleted in the del(13)(q12q22). Breakpoint positions are indicated by arrows

Both cellular angiofibromas had abnormal karyotypes that entailed heterozygous loss of material from the long arm of chromosome 13 (Table 1, Fig. 2), together with other chromosome aberrations (Table 1). In case 1, there was an unbalanced translocation between chromosomes 1 and 13 described as der(13)t(1;13)(q12 ~ 21;q14 ~ 21) accompanied by monosomy 14 and aberrations of chromosome 16 (Table 1, Fig. 2a and b). FISH analysis in case 1 showed deletion of the *RB1* probe in 111 out of 200 investigated interphase nuclei (Fig. 2c). In case 2, an interstitial deletion in chromosome 13 was found which was described as del(13)(q12q22) together with a balanced t(10;15)(p13;q22) (Table 1, Fig. 2d). FISH analysis of metaphase spreads showed that the *RB1* probe was heterozygously deleted also in case 2 (Fig. 2e and f).

## Discussion

The present study shows consistent heterozygous loss of material from chromosome arm 13q in two cellular angiofibromas, supporting the observation first put forward by Hameed et al. [9] that these tumors are (cyto)genetically similar to spindle cell lipomas. The examined tumors arose in the inguinoscrotal or paratesticular region and had structural aberrations of chromosome 13 affecting the q12-q22 bands. In both tumors, the loss of material from chromosome 13 was accompanied by other chromosome aberrations (Table 1). Also the only hitherto karyotyped cellular angiofibroma had loss of chromosome 13 together with additional chromosome aberrations [9]. Although the analyzed G-banded cases number only three, two of which are described here (Table 1), the data show that loss of 13q material is not (usually) the only cytogenetic anomaly in tumors of this type (Table 1, [9]). This, too, strengthens the cytogenetic similarity with spindle cell lipomas: Most of the 28 karyotypically abnormal spindle cell lipomas reported in the cytogenetic literature had loss of material from 13q together with other chromosome abnormalities, most often loss of or from 16q (http://cgap.nci.nih.gov/Chromosomes/Mitelman, Database last updated on August 11, 2016). Our analysis further showed that the structural aberrations affected bands 13q14~21 or 13q12-q22 which are also the most commonly lost in spindle cell lipomas [14–16]. The deletion of the *RB1* locus, found by FISH, adds evidence pointing in the same direction [7, 9, 11, 12]. Maggiani et al. [11] examined by interphase FISH two cellular angiofibromas. The first tumor had a deletion of 13q14 that included the *RB1* and *FOXO1* loci. In the second tumor, the deleted region contained *FOXO1* but not *RB1* [11].

Studies of spindle cell lipomas using FISH and single nucleotide polymorphism (SNP) array methodologies have identified two minimal deleted regions (MDR) in 13q14 [14, 15]. In MDR1, four genes are located, including the tumor suppressor gene *RB1*. In MDR2, there are 34 gene loci as well as the two microRNA genes miR-15a and miR-16-1 [14]. Because the expression levels of *RB1* were not significantly reduced and because no mutations were seen by sequencing, the authors concluded that there is no decisive support for *RB1* as the main target for the 13q deletions in spindle cell lipomas [14]. Instead, their data implicate miR-16-1 as a potential target for the 13q deletions in these tumors [14]. On the other hand, immunohistochemical staining for RB1 showed that nuclear RB1 expression was deficient in all examined spindle cell lipomas, pleomorphic lipomas, and cellular angiofibromas, as well as in 17 of 19 (89%) mammary-type myofibroblastomas [10]. At the moment, one cannot say more than that the molecular target of chromosome 13 aberrations in cellular angiofibromas remains unknown.

Although cellular angiofibroma overlaps morphologically with spindle cell lipoma and mammary-like myofibroblastoma, the three tumors also have some distinctive morphological features. In cellular angiofibroma, spindle-shaped cellularity is prominent as are hyalinized blood vessels and only minimal adipose tissue. Similarly, thick-walled blood vessels are not seen in the other two entities. In spindle cell lipomas, a mixture of mature adipocytes and bland spindle cells are found against a mucinous, myxoid, or fibrous background. In mammary-type myofibroblastoma, finally, fascicles of spindle cells with features of myofibroblasts are seen, with intervening hyalinized collagenous stroma together with various amounts of adipose tissue ([11, 12, 17] and references therein). The morphologic similarities of these tumors together with their cytogenetic similarities, in particular loss of material from the long arm of chromosome 13, suggest that they arise through largely identical pathogenetic mechanisms from a common stromal precursor cell which then undergoes (myo)fibroblastic or adipocyte differentiation [11, 17].

## Conclusions

Our results show consistent involvement of chromosome 13 in cellular angiofibromas with loss of 13q, underscoring the suggested (cyto)genetic similarity between cellular angiofibroma and spindle cell lipomas.

## Abbreviations

FISH: Fluorescence in situ hybridization
HE: Hematoxylin and eosin
MDR: Minimal deleted region
SNP: Single nucleotide polymorphism
